# Do Tapping and Massaging during Tourniquet Application Promote Dilation of Forearm Cutaneous Veins? A Pilot Quasi-Experimental Study

**DOI:** 10.3390/healthcare11040522

**Published:** 2023-02-10

**Authors:** Kae Yasuda, Kazunori Okada, Naotaka Sugimura, Rika Yano

**Affiliations:** 1 Graduate School of Health Sciences, Hokkaido University, Sapporo 060-0812, Hokkaido, Japan; 2 Department of Clinical Laboratory Sciences, Faculty of Health Sciences, Japan Healthcare University, Sapporo 062-0053, Hokkaido, Japan; 3 Faculty of Health Sciences, Hokkaido University, Sapporo 060-0812, Hokkaido, Japan

**Keywords:** nurse, peripheral intravenous catheterization, venous dilatation

## Abstract

Successful insertion of a peripheral intravenous catheterization requires that veins be sufficiently dilated. This study aimed to clarify the venous dilation effect of including tapping or massaging to the application of a tourniquet at the cutaneous veins of healthy adults’ forearms. This was a quasi-experimental study of 30 healthy adult volunteers. Each participant underwent all three venous dilation procedures, which included the tourniquet application (Control condition), the tourniquet application and tapping the participant’s forearm (Tapping condition), as well as the tourniquet application combined with massaging the participant’s forearm (Massage condition). To clarify the venous dilation effects, venous indices were measured, namely the venous diameter (mm), depth (mm), and palpation score. After applying all venous dilation procedures, the venous diameter and palpation score significantly increased. However, no significant difference was observed between the control condition and each intervention condition. The depth in the control and tapping conditions decreased significantly in contrast to the Massage condition. Moreover, a subgroup (nine participants with a venous diameter less than 3 mm after the control condition) had similar results. This study found that additional tapping or massaging after tourniquet application could be less effective in promoting dilation in the forearm veins of healthy adults. Future studies should examine the efficacy and effectiveness of venous dilation in a wide target population while considering intervention methods.

## 1. Introduction

Peripheral intravenous catheter (PIVC) insertion to administer infusion solution and antibiotics is a common medical procedure experienced by 80% of hospitalized patients [1]. Guidelines recommend placing the PIVC in the forearm cutaneous vein as opposed to the median cubital vein based on daily activities and the risk of complications [2,3]. However, the forearm cutaneous vein is thinner than the median cubital vein and branches peripherally [4]. Consequently, approximately 12% to 26% of patients undergo multiple insertions after initial failed PIVC insertion [5,6,7,8]. Multiple PIVC punctures not only carry the risk of complications such as pain, nerve damage, and arterial puncture [9] but the time and staffing required for needle use and puncture action are also costly [10]. Therefore, it is necessary to devise ways to make the initial insertion of a PIVC successful.

A PIVC is inserted by palpating veins to check they run and selecting a vein of sufficient thickness [11]. In recent years, it has been reported that guided puncture using an ultrasound device is also effective [12]. Insertion of a PIVC into a vein with a narrow lumen that is challenging to palpate is particularly difficult with either approach [13,14]. Therefore, it is necessary to dilate the vein sufficiently and select a suitable vein for PIVC insertion using venous palpation.

The most frequently performed venous dilation method comprises using a tourniquet to promote venous blood stasis [15]. However, solely using a tourniquet may not be sufficient to promote venous dilation. To apply a tourniquet, nurses instruct patients to open and close a fist as well as to grip a fist. Moreover, the nurses massage the forearm, tap the vein [16], and heat the forearm [17,18] of a patient to apply a tourniquet. Particularly, tapping and massaging are procedures in which the nurse applies stimulation to the patient using a proactive approach. Thus, it does not require the patient’s cooperation nor the preparation of supplies and can be performed in a short time and easily, regardless of the situation. Studies have reported that massaging a forearm can cause blood stasis at the puncture site [6,19]. Furthermore, over half of the 13 experienced and 8 expert nurses with a high success rate of PIVC insertion performed massaging in the selection of insertion sites in the indistinct veins of older adults [20]. Research also indicates that stimulating a target vein with light tapping (mechanical stress such as shear stress) promotes venous dilation by releasing nitric oxide (NO), releasing other venous dilators from the vein endothelium [21], and activating the axon reflex [22]. 

Tapping and massaging are manual procedures that have been verified by comparing venous sizes in the median cubital vein in healthy adults. Ichimura et al. (2015) measured the venous dilation effect of tapping or massaging on applying a tourniquet in healthy adults [23]. Specifically, the study included venous cross-sectional area and depth and compared the values to a control group that received only the application of a tourniquet. In contrast, Yasuda et al. (2019) verified the venous dilation effect of massaging a forearm performed by expert nurses based on their analysis of the massage movements to push blood up from the periphery to the center of the body [24]. The results showed a significant increase in the cross-sectional area as opposed to only applying a tourniquet. Thus, the previous study suggests that massage extracted from the performance of expert nurses with a high puncture success rate could also be effective as a venous dilation technique.

However, the venous dilation effect of tapping and massaging on the forearm cutaneous vein has not been clarified. Furthermore, previous studies on the median cubital vein are not necessarily applicable to the forearm vein regarding tapping and massage, nor is it clear whether effective venous dilation can be achieved during PIVC insertion. This could be caused by the validation following the venous dilation effect of heat application on the median cubital vein and forearm cutaneous vein. Yamagami et al. (2017) conducted a randomized controlled intervention trial using hot pack application on the forearm cutaneous veins in healthy adults and found that the venous diameter and cross-sectional area were significantly larger in the intervention group that received a 40 ± 2 °C hot pack applied to the forearm for 15 min before applying a tourniquet than in the control group that received only applying a tourniquet [25]. Particularly, heating a forearm promotes an increase in blood flow as the skin is stimulated to accept the heating stimulus of the heated product [19,26]. However, the change in the cross-sectional area before and after the intervention was smaller than in another study [27] that performed a similar intervention on the median cutaneous vein, although the detailed reasons are not clear [25]. Thus, the venous dilation effect on the median cubital vein is not necessarily transferable to the forearm cutaneous vein.

Therefore, this study aims to clarify the venous dilation effect of tapping and massaging extracted from expert nurses’ movements after the application of the tourniquet in forearm cutaneous veins of healthy adults in their 20s, compared with the venous size of a control group wearing only a tourniquet. Confirmation of these venous dilatation effects would assist in selecting the optimal vein for PIVC insertion by efficiently choosing among the many venous dilatation techniques available.

## 2. Materials and Methods

### 2.1. Study Design

This study used a quasi-experimental design. The participants underwent all three venous dilation procedures in a randomized order. The three venous dilation procedures evaluated were the tourniquet application (Control condition), the tourniquet application combined with tapping the forearm (Tapping condition), and the tourniquet application combined with massaging the forearm (Massage condition).

### 2.2. Participants

A convenience sample was used comprising volunteers aged between 20 and 29 years recruited from Japanese universities through posters and flyers. The age of the participants was restricted because research reports that aging affects venous responses [28]. This study excluded participants receiving treatment for cardiovascular disease or severe skin diseases and who had wounds or eczema on their forearms.

### 2.3. Sample Size

The sample size was set at 30 participants. There are no studies, to our knowledge, reporting the venous dilation effects of tapping or massage to the forearm cutaneous veins. Thus, in order to determine the sample size, it was calculated based on previous studies that reported the application of tapping or massage to the median cubital vein, including venous dilation in healthy, 20-something-year-old adults [23,24]. A sample of 30 participants was needed based on the difference in venous cross-sectional area between the control group (applying a tourniquet) and the intervention group (applying a tourniquet and other venous dilation procedures) after the intervention. A significance level of 0.5, a power of 0.80, and a paired *t*-test with an effect size of 0.5. and G*Power software version 3.1.9 (G*Power, Heinrich Heine University, Düsseldorf, Germany) were used for the sample size calculation. 

### 2.4. Experimental Environment

The study was conducted at a university in Japan between April and June 2018. All procedures and measurements were conducted in the same laboratory with temperature (22–24 °C) and humidity (45–65%) consistent with summer hospital standards. The participants were asked to assume a supine position and keep their forearms on the bed during the intervention. Particularly, the veins dilate more in a supine position than in a seated position [29].

### 2.5. Determining the Target Vein

The current study assumed that PIVC insertion would be performed using a 22 gauge catheter for infusion; thus, no actual puncture was performed. Recommendations stipulate that medical professionals ought to perform venipuncture in the patient’s non-dominant arm to reduce the associated morbidity [10]. Accordingly, the non-dominant arm of each participant was used to determine the target vein. Ultrasonography was used to determine the target veins (Aplio XV, 12L probe, Canon medical systems, Otawara, Japan), and the target vein was defined as follows: (1) ≤30 mm distal from the antecubital fossa but ≤120 mm proximal to the radial styloid, and (2) as peripheral as possible. Notably, only the cephalic, median, or basilica veins ≥ 2.5 mm in a straight line, lying ≤ 10 mm deep, and with a diameter ≥ 0.9 mm could be used in this study [2,3,25,30]. Finally, the target vein and site of the tourniquet application were marked to ensure measurement at the target site.

### 2.6. Experimental Procedure

**Figure 1:**
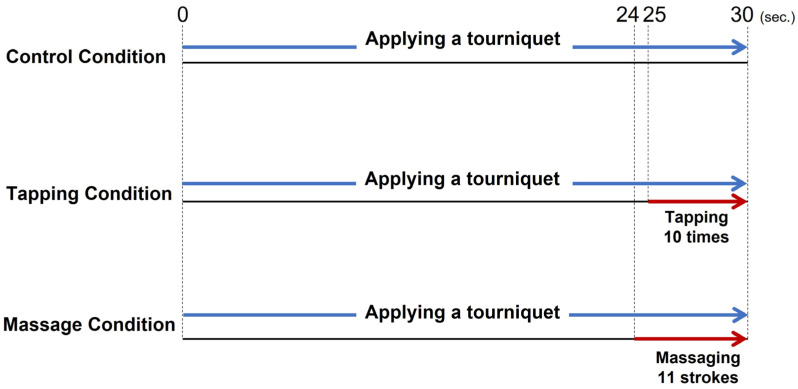
Protocol of venous dilation procedures. Notes: Control condition, the tourniquet application; Tapping condition, the tourniquet application and to be tapped on the forearm; Massage condition, the tourniquet application and to be massaged on the forearm. All three venous dilation procedures were performed for all participants.

Each venous dilation procedure is illustrated in Figure 1. The participants underwent all three venous dilation procedures in a randomized order with a 15 min washout period between interventions. To ensure consistent pressure applied to the forearm, two nurses with at least five years of clinical experience performed tapping and massaging before measuring the pressure applied (SR soft vision, numeric value version, Sumitomo Riko, Aichi, Japan). The mean values were calculated as 10 ± 3 mmHg and 40 ± 5 mmHg for tapping and massaging, respectively.

The same researcher performed each technique. Sufficient training was provided to ensure consistent tapping and massaging pressures within the range of the average values. The participants were requested to avoid clenching their fists.

#### 2.6.1. Tourniquet Application

A tourniquet was applied 10 cm above the target vein using a rubber-type tourniquet with a scale (tourniquet with scale, TTQ-100-1, TAIYO Instruments INC, Osaka, Japan) to ensure that a constant of approximately 75 mmHg pressure was applied for 30 s [31,32]. Applying a tourniquet at 60–80 mmHg for 30 s can promote venous dilation [33].

#### 2.6.2. Tapping the Vein

In this study, “Tapping” was defined as lightly touching the skin over the target vein with the practitioner’s dominant index and middle finger. Tapping was performed 10 times during the last 5 s of applying a tourniquet [23,34].

#### 2.6.3. Massaging the Forearm

“Massaging” referred to the act of applying a stroking motion from the wrist to the forearm of the participant using the palm of the researcher’s dominant arm. Massaging was performed 11 times during the last 6 s of applying a tourniquet.

### 2.7. Data Collection

**Figure 2:**
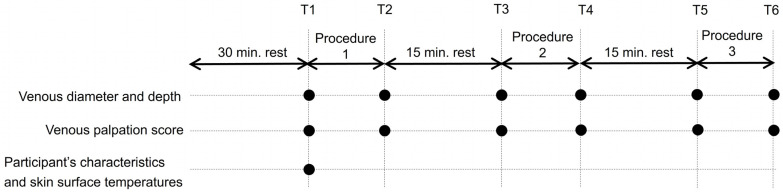
Study protocol. Notes: (●) Measurement points. T1, at the start of the intervention; T2, after the first venous dilation procedure; T3 and T5, after 15 min of rest; T4, after the second venous dilation procedure; T6, after the final venous dilation procedure.

This study collected data at six points: at the start of the intervention (T1), after the first venous dilation procedure (T2), after 15 min of rest (T3), after the second venous dilation procedure (T4), after 15 min of rest (T5), and after the final venous dilation procedure (T6; Figure 2). Specifically, each participant entered the measurement room, was placed in a supine position on the bed, and spent 30 min at rest, including during orientation.

#### 2.7.1. Participants’ Characteristics

The characteristics of the participants were assessed before selecting the target veins, namely sex, age, weight, height, body mass index (BMI), as well as body temperature, blood pressure, and pulse.

#### 2.7.2. Venous Diameter and Depth

**Figure 3:**
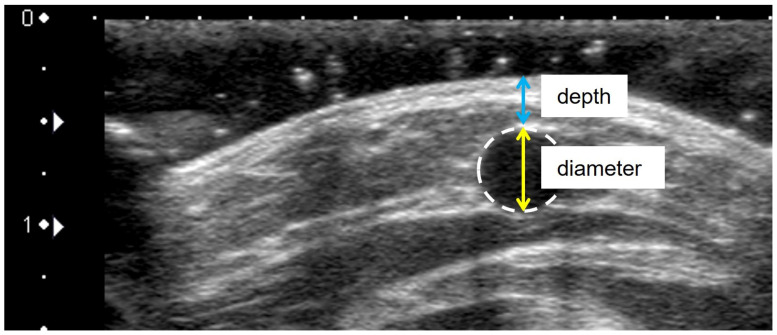
Measurement of venous diameter and depth by ultrasonography. Notes: Venous diameter (mm) was measured from the upper end to the lower end of the vein. Depth (mm) was measured as the vertical distance from the skin surface to the apex of the vein circle.

To evaluate venous dilation of procedures, the venous diameter and depth were measured as the primary and secondary indexes, respectively [35]. Ultrasonography was used to measure venous diameter and depth at all measurement points (T1–T6). A cross-sectional B-mode image of the vein of interest was acquired while ensuring that the probe did not compress the vein. Furthermore, the diameter (mm) was measured from the upper end to the lower end of the vein (Figure 3), while the depth (mm) was measured as the vertical distance from the skin surface to the apex of the vein circle (Figure 3). The measurements were conducted by two researchers to confirm validity and reproducibility. Researcher A was a registered nurse trained in the use of ultrasonography. Similarly, researcher B had substantial experience in ultrasonography and was a registered medical sonographer certified by the Japan Society of Ultrasonics in Medicine. The measurements by the two researchers demonstrated high inter-rater reliability (ICC (2.1), venous diameter: 0.99, and depth: 0.99). However, only the measurement data from researcher A were used in the analysis. Moreover, the intra-rater reliability of the venous diameter and depth measurements made primarily by researcher A were analyzed. The results showed high reliability (ICC [1.1], venous diameter: 0.92, and depth: 0.99) [36].

#### 2.7.3. Skin Surface Temperature

A thermographic image of the forearm and palmar skin surface temperature at T1 was obtained using thermography (R300, NEC, Tokyo, Japan).

#### 2.7.4. Venous Palpation Score

Kato’s venous palpation score (0: impalpable, 1: slightly palpable, 2: less palpable, 3–4: more palpable, 5–6: well palpable) was altered by recategorizing the scale into three steps (0: impalpable, 1: slightly palpable, 2: sufficiently palpable) [37]. To simplify evaluations, “3–4: more palpable” of Kato’s venous palpation score was included in “1: slightly palpable” in this recategorized scale. The same researcher determined the venous palpation score at T1 through T6.

### 2.8. Data Analysis

All results were reported as the mean (standard deviation), number (%), or 95% confidence interval. The change ratios of venous diameter and depth were calculated as (measurement post venous dilation procedure)/(measurement pre-venous dilation procedure) × 100. The venous diameter and depth were compared between the cephalic vein and median or basilica veins using the Student’s *t*-test. This study used paired *t*-tests to compare the results of venous diameter and depth before and after the interventions. Furthermore, the change ratio of venous diameter or depth between each intervention condition and control condition was compared using a linear mixed-effects model that added a random effect of an individual (one-way repeated-measures analysis of variance (ANOVA)) before performing Dunnett tests. Moreover, Fisher’s exact test was used to compare the proportion of participants classified by a change in score among conditions.

PIVC insertion is challenging when a vein diameter is less than 3 mm [11]. However, tapping has also been reported as an effective method for the median cubital vein, which has a small cross-sectional area that is difficult to palpate [34]. Thus, participants with thin veins (<3 mm) after the control condition were selected, and the effect of each venous dilation procedure was analyzed using the same methods as a subgroup analysis. In addition, these participants’ characteristics were compared with participants who have thick veins (≥3 mm) using Student’s *t*-tests or a Chi-square test.

The InfReC Thermography Studio 5.1 advanced thermal image analysis software (Nippon Avionics, Tokyo, Japan) and JMP Pro software, version 13.0 (SAS Institute Inc., Cary, NC, USA) were used for data analyses. This study considered *p* ≤ 0.05 as statically significant. Furthermore, Cohen d was used as the effect size in the Student’s *t*-test. Partial η^2^ was also used in ANOVA and φ was used in the Chi-square test [38,39].

### 2.9. Ethical Considerations

The following details were explained to the participants: (1) participation in the study was voluntary and no consequences would arise for withdrawing from the study, (2) all data would be recorded anonymously and de-identified, and (3) all data would remain protected for at least five years to ensure confidentiality. Each participant provided their signed, informed consent. This study was approved by the ethics committee of the Hokkaido University Faculty of Health Sciences (registered number 17-119, 3 April 2018) and was conducted in accordance with the Declaration of Helsinki.

## 3. Results

All 30 healthy volunteers (15 men and 15 women) participated in this study. As no significant difference was observed in the venous diameter and depth between the cephalic, median, and basilic veins at T1 (diameter: *p* = 0.425, depth: *p* = 0.121, Table 1), subsequent analyses were performed by integrating the vein types.

### 3.1. Change in Venous Diameter and Depth for Each Venous Dilation Procedure

Table 2 illustrates that venous diameters increase significantly after the procedures (*p* < 0.001). However, the after-venous diameter did not differ significantly between each intervention and control condition (Control condition vs. Tapping condition: *p* = 0.864, Control condition vs. Massage condition: *p* = 0.337). The change ratio in venous diameter before and after each procedure was approximately 120%. Furthermore, no significant differences in venous depths were observed after the procedures between each intervention and control condition (Control condition vs. Tapping condition: *p* = 0.994, Control condition vs. Massage condition: *p* = 0.761). However, only the control and tapping conditions were significantly shallower than before the procedure (*p* < 0.001).

### 3.2. Venous Palpitation Score and Associated Change

In contrast to the pre-conditions, Table 3 shows that the percentage of higher venous palpation scores increase significantly post intervention in all conditions (all conditions, *p* < 0.001). However, no statistically significant difference was found in the percentage of participants scoring after each condition (*p* = 0.680).

### 3.3. Subgroup Analysis: Venous Dilation Effect of Each Procedure in the Thin Vein Group

This study consisted of nine participants with a venous diameter value less than 3 mm after the control condition implementation (i.e., thin vein group). These participants tended to have palm skin surface temperatures that were approximately 1 °C lower than the 21 participants with venous diameters of 3 mm or greater after the control condition (*p* = 0.056, d = 0.794, Table 4).

**Figure 4:**
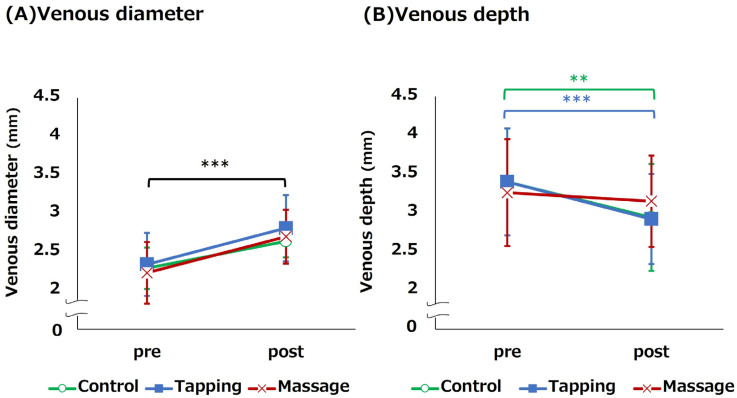
Venous dilation effects in thin vein group (n = 9). Notes: Control, Control condition; Tapping, Tapping condition; Massage, Massage condition; paired *t*-test, ** *p* < 0.01 *** *p* < 0.001 ((**A**), all conditions were significantly larger between pre and post; (**B**), Control and Tapping conditions were significantly larger between pre and post); linear mixed-effects model (one-way repeated-measures analysis of variance (ANOVA)) and Dunnett test. The bars at each time point indicate 95% confidence intervals.

The venous diameters after the procedures were significantly greater than before the procedures in all three conditions (all conditions, *p* < 0.001). However, the lower bound of the 95% confidence interval for the venous diameter after the procedures did not exceed 3 mm in any condition (Figure 4). Furthermore, no statistically significant difference was observed in the change in venous diameter before and after the intervention between the control condition and each intervention condition (Tapping condition: *p* = 0.772, Massage condition: *p* = 0.772), which exhibited a small effect size (Partial η^2^ = 0.006; Figure 4). Moreover, the venous depth was significantly shallower before and after the procedure only in the control and tapping conditions (Control condition: *p* = 0.002, Tapping condition: *p* < 0.001; Figure 4).

## 4. Discussion

This comprises the first pilot study to verify the venous dilation effect and efficacy of expert nurses tapping and massaging the forearm cutaneous vein, which is the recommended site for PIVC insertion. In previous studies, venous dilation was observed in the median cubital vein in healthy adults with additional tapping and massaging when applying a tourniquet [23,24]. However, this study found no significant difference in the change in venous diameter between each intervention condition and the control condition, which applied only a tourniquet. In contrast to a study by Yasuda et al. (2019), the amount of change in the venous cross-sectional area was approximately 5.5 to 7.2 mm^2^ in this study despite using similar interventions on the median cubital vein [24]. This result confirms that the change in the cross-sectional area was 1.6 to 3 times greater than the change in venous diameter converted to the cross-sectional area (estimated at 2.3 to 3.3 mm^2^) in the venous diameter in the present study. Therefore, additional tapping or massaging extracted from expert nurses’ movements after the application of the tourniquet could be less effective in promoting dilation in the forearm veins of healthy adults in their 20 s. 

Consistent with Ichimura et al. (2015) and Yasuda et al. (2019), this study included healthy adults with a standard physical build and set the same conditions for each participant’s age, gender ratio, as well as the environmental temperature and humidity [23,24]. Despite using as few confounding factors as possible, the results of the current study differed from previous studies. These findings could be attributed to differences in the site of the vein where the interventions were performed. The venous depth of forearm cutaneous veins at pre-intervention measured in this study (3.29–3.41 mm) was approximately 1 mm deeper than the median cubital vein measured in these two previous studies (Ichimura et al. (2015): 1.60–1.80 mm, Yasuda et al. (2019): 2.10–2.30 mm) [23,24]. Thus, it is unlikely that tapping to stimulate the veins or massaging to promote venous blood stasis manually [6,19] will produce a sufficient response to promote venous dilation in the deeper forearm veins. Particularly, the forearm cutaneous veins are located in a flatter, peripherally bifurcated area as opposed to the central portion of the cubital vein, which is located in the flexure [4]. Thus, this research suggests that massaging pushes blood up from the periphery to the center of the measurement arm in the cutaneous veins, which reduces the likelihood of changing its diameter and depth. The possibility that vein type may be a confounding factor should be considered when examining the efficacy of venous dilation procedures in the future.

Studies show that adult patients with a venous diameter of less than 3 mm result in difficulty inserting PIVC after applying a tourniquet [11], necessitating additional venous dilation techniques. The current study also observed a subgroup analysis in the nine patients with a venous diameter less than 3 mm after the control condition and who required additional venous dilation procedures. However, no statistically significant differences were found between each intervention condition and the control condition, and the effect sizes were small [38]. In efficacy terms, the lower limit of the 95% confidence interval for venous diameter after each intervention did not exceed 3 mm. The results imply that additional tapping or massaging may not be sufficient to achieve effective venous dilatation for PIVC insertion. Particularly, these participants were characterized by a palmar skin surface temperature that was approximately 1 °C lower than that of the other participants. As peripheral coldness is associated with a lack of blood flow [40], it is assumed that the amount of blood flowing initially to the forearm was low and that manual intervention alone could have been inadequate. In the future, it will be necessary to evaluate the venous dilation effect using additional techniques other than these for adult participants with veins smaller than 3 mm in diameter who have difficulty inserting PIVCs. Alternatively, tapping and massaging methods used in this study might have been insufficient to promote effective venous dilation of the forearm cutaneous veins. However, these venous dilation procedures were conducted using the same methods as previous studies. Notably, excessive intensity or frequency of interventions could pose a risk of skin problems, such as internal bleeding in older adult patients with fragile skin or patients lacking blood coagulation factors due to disease or treatment. Thus, future studies ought to carefully consider safe and effective methods in this regard.

The current study had several limitations. First, it is difficult to generalize the results of this study because the participants, recruited as a convenience sample, were limited to healthy adults in their 20s with a standard physical build. However, the characteristics and reactivity of veins vary with disease, treatment, physical build, and age [41]. Thus, future studies should examine the efficacy and effectiveness of venous dilation in a wide target population while considering intervention methods. Second, there might have been a lack of power in the subgroup analysis. However, the venous diameters in the control and each intervention group exhibited small effect sizes. Finally, some of the tapping and massaging methods performed in this study are being used in clinical practice. Thus, future research should verify the effectiveness of different tapping and massaging methods such as applying pressure and using fingers.

## 5. Conclusions

This pilot study examined the venous dilation effect of tapping and massaging extracted from expert nurses’ movements after applying a tourniquet to the forearm cutaneous veins of healthy adults in their 20s. Consequently, no statistically significant differences were found in venous diameter or palpability between the control condition and each intervention condition. Furthermore, similar results were obtained in participants with a venous diameter of less than 3.0 mm after the control condition (i.e., only applying a tourniquet). Moreover, the lower limit of the 95% confidence interval for the venous diameter after two intervention conditions did not exceed 3.0 mm, which is considered sufficient for PIVC insertion. Therefore, in the selection of veins for PIVC insertion in a healthy adult’s forearm, only applying a tourniquet may be sufficient and it may be unnecessary to forcibly add tapping or massage. If it is impossible to find sufficiently large veins optimal for PIVC insertion with the use of a tourniquet alone, it may be necessary to combine these techniques with heat application or other venous dilation techniques. Therefore, future studies should examine the efficacy and effectiveness of venous dilation in a wide target population while considering intervention methods.

## Figures and Tables

**Table 1:** Participants’ characteristics (n = 30).

Characteristic	n (%)	Mean (SD)
Sex		
Men	15 (50.0)	-
Women	15 (50.0)	-
Dominant forearm		
Right	26 (86.7)	-
Left	4 (13.3)	-
Target vein		
Cephalic	12 (40.0)	-
Median	17 (56.7)	-
Basilic	1 (3.3)	-
Age (years)	-	22.47 (1.25)
BMI (kg/m^2^)	-	21.44 (2.29)
Body fat percentage (%)	-	21.16 (7.84)
Body temperature (°C)	-	36.40 (0.29)
Pulse (beats/min)	-	60.73 (9.13)
Systolic BP (mmHg)	-	108.87 (11.14)
Diastolic BP (mmHg)	-	58.60 (9.13)
Skin surface temperature on forearm (°C)	-	33.07 (1.04)
Skin surface temperature on palm (°C)	-	33.55 (1.81)
Venous diameter	-	
Cephalic venous diameter at T1 (mm, n = 12)		2.53 (0.59)
Median and basilic venous diameter at T1 (mm, n = 18)		2.67 (0.31)
Venous depth	-	
Cephalic venous diameter at T1 (mm, n = 12)		3.91 (1.77)
Median and basilic venous diameter at T1 (mm, n = 18)		3.04 (1.19)

Abbreviations: BMI, body mass index; BP, blood pressure; SD, standard deviation; T1, at the start of the intervention.

**Table 2:** Change in venous diameter and depth (n = 30).

	Pre	Post	*p*	Change Ratio
	Mean (SD)			Mean (SD)
Venous diameter (mm)				
Control condition	2.60 (0.43)	3.32 (0.60)	<0.001 ^a^	128.45 (16.78)
Tapping condition	2.63 (0.52)	3.25 (0.50)	<0.001 ^a^	126.41 (22.48)
Massage condition	2.61 (0.64)	3.13 (0.61)	<0.001 ^a^	124.36 (31.23)
*p*	0.980 ^b^	0.432 ^b^		
Partial η^2^	<0.001	0.019		
Venous depth (mm)				
Control condition	3.41 (1.46)	3.03 (1.27)	<0.001 ^a^	89.96 (10.37)
Tapping condition	3.41 (1.48)	3.00 (1.27)	<0.001 ^a^	90.38 (15.31)
Massage condition	3.29 (1.39)	3.23 (1.34)	0.528 ^a^	99.95 (15.48)
*p*	0.926 ^b^	0.743 ^b^		
Partial η^2^	<0.001	<0.001		

Abbreviations: SD, standard deviation. Notes: ^a^ paired *t*-test; ^b^ linear mixed effect model (one-way repeated-measures analysis of variance (ANOVA)) and Dunnett test; partial η^2^: small (η^2^ = 0.01), medium (η^2^ = 0.06), large (η^2^ = 0.14).

**Table 3:** Change in venous diameter and depth (n = 30).

	Venous Palpation Score	Pre	Post	χ^2^/*p* df
		n (%)		
Control condition	0: impalpable	14 (46.67)	0 (0.00)	35.40/<0.001 ^a^ df-2
	1: slightly palpable	16 (53.33)	10 (33.33)	
	2: sufficiently palpable	0 (0.00)	20 (66.67)	
Taping condition	0: impalpable	15 (50.00)	1 (3.33)	30.44/<0.001 ^a^ df-2
	1: slightly palpable	15 (50.00)	11 (36.67)	
	2: sufficiently palpable	0 (0.00)	18 (60.00)	
Massage condition	0: impalpable	17 (56.67)	2 (6.67)	32.39/<0.001 ^a^ df-2
	1: slightly palpable	13 (43.33)	9 (30.00)	
	2: sufficiently palpable	0 (0.00)	19 (63.33)	
χ^2^/*p*		0.623/0.733 ^a^	2.305/0.680 ^a^	
df		df-2	df-4	

Notes: df, degrees of freedom; ^a^ Fisher’s exact test.

**Table 4:** Differences in participants’ characteristics between venous sizes (n = 30).

Characteristic	Thin Vein Group (n = 9)	Thick Vein Group (n = 21)	*p*	φ
	n (%)			
Sex				
Men	3 (33.33)	12 (57.14)	0.427 ^a^	0.220
Women	6 (66.67)	9 (42.86)		
Venous palpation score post Control condition				
0: impalpable	0 (0.0)	0 (0.0)	0.431 ^a^	0.154
1: slightly palpable	4 (44.44)	6 (28.57)		
2: sufficiently palpable	5 (55.56)	15 (71.43)		
	**Mean (SD)**		***p***	**d**
BMI (kg/m^2^)	20.88 (1.39)	21.69 (2.57)	0.382 ^b^	0.354
Body fat percentage (%)	21.73 (6.56)	20.92 (8.47)	0.800 ^b^	0.102
Body temperature (°C)	36.36 (0.22)	36.41 (0.33)	0.629 ^b^	0.195
Pulse (beats/min)	61.89 (8.75)	60.24 (9.45)	0.671 ^b^	0.178
Systolic BP (mmHg)	103.78 (8.74)	111.05 (11.62)	0.102 ^b^	0.673
Diastolic BP (mmHg)	61.89 (7.46)	57.19 (10.41)	0.232 ^b^	0.486
Skin surface temperature on the forearm (°C)	32.61 (1.13)	33.28 (0.95)	0.110 ^b^	0.658
Skin surface temperature on the palm (°C)	32.59 (2.36)	33.96 (1.40)	0.056 ^b^	0.796
Venous diameter at post Control condition (mm)	2.64 (0.28)	3.61 (0.44)	<0.001 ^b^	1.805
Venous depth at post Control condition (mm)	3.23 (1.26)	2.94 (1.29)	0.574 ^b^	0.226

Abbreviations: BMI, body mass index; BP, blood pressure; SD, standard deviation. Notes: ^a^ Chi-square test; ^b^ Student’s *t*-test; φ, small (φ = 0.10), medium (φ = 0.30), large (φ = 0.50), d: small (d = 0.20), medium (d = 0.50), large (d = 0.80).

## Data Availability

Data sharing not applicable.
